# The Chicago Pathological Society

**Published:** 1882-02

**Authors:** 


### ARTICLE VII.

THE CHICAGO PATHOLOGICAL SOCIETY. Stated Meeting held at the President's residence, Nov. 28, 1881.

Dr. Henry M. Lyman, President, related a remarkable case of

### RUPTURE OF THE HEART.

The patient, a man sixty-two years old, had had several attacks of cerebral rheumatism, but no acute attack of the disease. He was rather plethoric. Last summer he suffered from dyspnœa and syncope at various times. During the autumn he went shooting ducks, but a paroxysm obliged him to return, and he felt a severe pain in his chest. At one A. M. severe paroxysms set in, and seemed to arise from the stomach. There was no angina pectoris. An hypodermic injection of ¼ gr. of morphine was given, and partly relieved the pain. Before morning the patient got up, and while he sat on the chamber-pot, he fell over dead.

*Post-mortem.*—Thorax and abdomen looked normal. As soon as the pericardium was laid open a heart clot came into view. The cardial muscle was degenerated. The left ventricle was rent an inch in length near the septum. Laying open the ventricle, there was no atheroma, nor valvular disease. The portion of the clot nearest the rent was white, and gave evidence of being older than the rest. The exertion at the hunting ground must have been the first step in laceration, which afterward continued; the effort at defecation having proven the last cause of the rupture.

*Remarks.*—The general impression is, that rupture of the heart must necessarily be sudden, and cause instantaneous death. In this case, life must have been prolonged about fifteen hours after the beginning of the injury. The cavity of the pericardium was pretty well filled, though not distended. The walls of the heart at the point of rupture were rather thick. This case was one of fatty degeneration, and the heart was overloaded with fat besides. Fatty degeneration of the walls of the heart, the doctor remarked, was often met with after death, especially from anæsthetics; yet death did not always take place, and, as the disease had no symptoms, it was almost impossible to be aware of it before an autopsy was made.

### GUBLER'S THERAPEUTICS.

Dr. H. M. Lyon reviewed orally the late American edition of Gubler's Therapeutics. This work has recently been translated. The author had one of those well-developed minds which had given the subject the most careful study. He had made a very thorough study of botany, and his works were numerous, especially in physiological chemistry, and allied sciences. He had been connected with many important societies, and his works had often been rewarded by medals and prizes. His book was especially valuable to learned practitioners, and he had carried the study of the subject to its higher laws.

The arrangement of division was not very well marked, however. The elimination of medicine was highly considered, and a large part of the therapeutical effects of drugs was referred to the change brought on by elimination.

The tolerance of drugs was also treated of at length in regard to tobacco, arsenic, opium, etc. The antagonism of medicines was treated briefly and in an able manner; and Gubler denied the fact of idiosyncrasies, except as something rare. We are now in the midst of a great change in chemical nomenclature. Organic compounds, as gluesides, hydro-carbons, etc., give us many intricate compounds. Hence, in the forming of alkaloids, it was doubtful whether the resultant compound always partook of the elements in its therapeutical workings. Such instances as the bromide of quinia were considered.

As to the classification of medicines, Gubler reviewed several; said it was impossible to make an absolute classification, and proposed a physiological one which was not any better. He illustrated the correlation of forces in the use of coffee, opium, etc., which prevented an excessive expenditure of forces. These medicines add more force to the system, as it were. Ainstie had made the same remarks in regard to the tonics, which are different from stimulants.

The hypodermic use of aconitia, bromo-hydrate of quinia and of jaborandi had been introduced or generalized through Gubler's works, and the bromo-hydrate of quinia never gave rise to any abscess, nor to cinchonism.

Dr. H. M. Lyman was conversant with the book. It was full of suggestive ideas, some rather old, yet every one became interested in its reading.

